# Supplementary material for: Are carbon emissions trading and green financial instruments synergistic? -Comprehensive quantitative research based on content analysis
Source: PLoS One. 2024 Mar 7;19(3):e0298601. doi: 10.1371/journal.pone.0298601 (PMC10919601; doi:10.1371/journal.pone.0298601)
Supplement: S1 Data — (ZIP) [file pone.0298601.s001.zip › Data/地级市Z_F工作报告.zip!/║╙─╧/║╫▒┌╩╨/└┤╫╘╣1⁄2╓┌║┼í╛┬φ┐╦╩2╛▌═°í┐_2004.docx]

各位代表：

　　本届政府1999年1月就职，任期即将结束。现在，我代表市人民政府，向大会报告过去五年的工作，对今后五年、重点是今年的工作提出建议，请予审议，并请政协各位委员提出意见。

　　一、过去五年工作回顾

　　五年来，本届政府在省委、省政府和市委的正确领导下，团结带领全市人民，认真实践“三个代表”重要思想，坚持发展第一要务，锐意进取，扎实工作，圆满完成了市七届人大历次会议确定的目标任务。

　　过去的五年，是我市国民经济持续健康发展，综合实力显著提高的五年。全市生产总值由1998年的73.23亿元增加到2003年的122.28亿元，年均增长11%。三次产业比例由24.4∶47.9∶27.7调整为19.5∶52.1∶28.4。财政一般预算收入由2.81亿元增加到4.40亿元，年均增长11.2%，其中市级由1.23亿元增加到2.27亿元，年均增长13.8%。金融机构商业贷款累计增加18亿元。资源型工业基地建设取得重要进展，先进制造业基地建设扎实起步，食品加工业基地建设形成规模与特色，工业经济质量效益明显提高。规模以上工业企业增加值由21.7亿元增加到41.8亿元，年均增长13.8%；实现利润由亏损4749万元扭转为盈利2.71亿元；经济效益综合指数由58.03提高到105.14，创历史最高；累计完成技改投资16.5亿元。鹤煤、万和、豫鹤、天海、天元等企业进一步发展壮大，“天海”牌汽车插接器、“大用”牌鸡肉制品等知名品牌的市场竞争力明显提高。农村经济全面发展。畜牧业优势进一步增强，2003年畜牧业产值24.9亿元，占农业总产值的58.3%，比1998年提高13.9个百分点；畜牧加工业产值25.6亿元，增长4.33倍；农民人均纯收入中来自畜牧业及相关行业的比重达到35.5%，提高6.1个百分点。全市农业总产值、增加值和农民人均纯收入增速连年居全省先进位次。优质小麦种植面积由4万亩增加到80.5万亩，出口实现零的突破。农业产业化水平继续提高，一批农业龙头企业发展壮大，辐射带动全市40%的农户受益。国家食品安全综合示范区项目正式启动。农产品质量安全水平全面提高。旅游景区景点累计实现门票收入2672万元，年均增长48%。

　　过去的五年，是我市重点项目建设取得历史性突破，基础设施进一步完善的五年。全社会固定资产投资累计完成111亿元，是上个五年的1.6倍，年均增长12%。仅2003年开工建设的项目中，投资超千万元的就达到60个，其中超亿元的12个，总投资规模61.6亿元。水泥厂、电厂项目以前所未有的力度和速度推进。投资2亿元的水泥厂二期工程竣工点火，投资4亿元的三期工程开工建设，四期工程前期工作正在积极进行。四期工程建成后，我市水泥年生产能力将达到540万吨，成为全省最大的新型干法水泥生产基地之一。投资22亿元的电厂二期工程进展顺利，投资近50亿元的三期工程通过不懈努力，有望今年上半年开工建设。投资10亿元的热电联供项目开工。这些电力项目建成后，全市发电装机容量将超过250万千瓦，成为全省重要的火力发电基地之一。总投资9.98亿元的盘石头水库开工建设，累计投资7.8亿元，已经具备下闸蓄水条件。采煤沉陷治理项目通过最后评审，争取国家资金3.6亿元。公路建设累计投资9.7亿元，新建、改建县级以上公路262公里，在全省率先实现了“村村通油路”，公路密度居全省第五位。电网改造投资6.8亿元，城乡供电条件显著改善，城乡同网同价工作走在全省前列。城市基础设施建设累计投资6.5亿元，老区污水处理厂正式运营，新区污水处理厂开工建设。新增节水灌溉面积25万亩，治理水土流失面积288平方公里。

　　过去的五年，是我市各项改革不断深化，对外开放特别是招商引资实现重大突破的五年。国有企业改革取得显著成效，95%的市属国有企业完成改制，35户企业破产终结，解决了一批沉积多年的难题，增强了企业活力和发展后劲，提高了经济效益。把国有企业改革与招商引资紧密结合，成功引进了新飞集团等一批国内外有实力的战略投资者。非公有制经济快速发展，增加值占全市生产总值的比重提高到44%。行政审批制度改革不断深化，市、县两级行政服务中心已经建立，行政审批事项精简比例达92%，居全省首位。财政改革全面推进，初步构建了公共财政框架，农村税费改革、粮食流通体制改革取得明显成效。社会事业、城市建设和管理体制、投融资体制等改革取得积极进展。招商引资氛围日益浓厚，工业、农业、社会事业、城市基础设施和公用事业、现代服务业等方面的招商引资都取得积极进展。累计实际利用境外资金4198万美元，出口创汇1.68亿美元。累计争取上级资金27亿元，为缓解我市经济社会发展资金紧张状况发挥了重要作用。[Page]

　　过去的五年，是我市城乡面貌发生很大变化，城市形象和品位提升最明显的五年。新区经国务院批准成为我市的新市区，争取到了“25+1”特殊优惠政策，被列为省重点支持城市，新区建设以更快速度、更高标准全面展开。累计完成固定资产投资37亿元，修建城市道路32公里，新区框架拉大到30平方公里，建成区面积15.2平方公里，人口10万人。新区绿化进一步上档次、上水平，新增绿地406万平方米。新增供热98万平方米。启动了工业园区、高教园区、外向型批发市场建设，产业支撑能力逐步增强。天滨、天泰等一批市场建成投用。成功创建了中国优秀旅游城市。淇河综合开发扎实启动。体育、文化等公用设施进一步完善，投资2000万元的游泳馆开工建设，投资近3000万元的图书馆、博物馆、群艺馆“三馆合一”的文化中心主体工程完工。建成区村庄改造正式启动。新区成为全省城市建设的亮点，为我市经济社会发展打造了良好平台。老区建设积极推进，群众生产生活条件逐步改善，城市活力进一步增强。山城区对长风路等5条主次干道进行了改、扩建，西环线开工建设，枫岭公园、污水管网、燃气管网改造取得积极进展。鹤山区鹤山街、鹤壁集南大街改造完毕。投资1.3亿元的新老区快速通道开工建设。县城和重点镇建设取得新成绩，善堂、高村、庞村被省政府确定为重点镇。建立了规范的国有土地储备出让制度，实行了城市公用事业特许经营。在环卫、绿化、园林等方面引入竞争机制。全市城镇化率达到41%，比1998年提高12.7个百分点。

　　过去的五年，是我市科技教育快速发展，社会事业全面进步的五年。科技、教育、文化、卫生、体育累计支出11.4亿元，年均增长17.8%。科教兴鹤战略深入实施，科技开发和创新能力不断增强，取得国家级奖励成果1项、省部级奖励成果17项，一大批先进适用技术得到推广，科技对经济增长的贡献率达到41%。“两基”工作成绩突出，小学儿童入学率达到99.99%，小学毕业生升学率达到99.2%，受到国家和省表彰。市区及县城基本普及了高中阶段教育，普通高中招生人数和高招上线人数比1998年翻了一番。高等教育、职业教育取得长足发展，成立了职业技术学院，现已初具规模，显现特色，两个专业被教育部列为重点专业。新区教育和文化基础设施日益完善，老区教育基础设施得到进一步改善。农村中小学危房改造工作扎实推进。广场文化、社区文化、文化下乡活动蓬勃开展，一批剧目荣获重要奖项，文化市场整顿力度加大，文物保护管理不断加强。初步建立了突发公共卫生事件应急救治机制，加强了市、县、乡三级卫生保健网络和传染病防治体系建设，开始实施新一轮农村初级卫生保健规划，开展了新型农村合作医疗试点。全民健身活动广泛开展。广播电视综合覆盖率达到100%，数字化进程大大加快。人口和计划生育工作继续加强，人口自然增长率由8.31‰下降到4.23‰。资源开发利用水平进一步提高。生态建设和环保工作扎实推进，森林覆盖率由15.5%提高到18.6%，淇河水质常年保持在二类以上。双拥和军民共建工作扎实有效，其他工作也取得了新成绩。

　　过去的五年，是我市城乡居民收入不断增长，人民生活水平显著提高的五年。把不断提高人民生活水平作为政府工作的出发点和落脚点，高度重视解决群众特别是困难群众的生产生活问题。2003年，城镇居民人均可支配收入达到5977元，农民人均纯收入达到2554元，分别比1998年增长58.4%和33.6%。城乡居民消费水平和生活质量进一步提高。社会消费品零售总额累计完成121.8亿元，年均增长10.3%。城镇和农村居民恩格尔系数分别由41.8%和52.3%下降到33.8%和46.5%。城镇居民人均住房使用面积由10.5平方米提高到17.4平方米，农村居民人均住房面积由23.6平方米提高到31.4平方米。城市绿地率达到24%，绿化覆盖率达到31%。互联网用户达到5.6万户，固定电话用户达到23.1万户，移动电话用户达到19.7万户。建立了涵盖养老、失业、低保、医疗等方面的社会保障体系，建成了街道、社区劳动保障服务平台。克服就业容量小、安置任务重等困难，累计新增城镇就业3.52万人，安置国有企业下岗失业人员2.46万人，再就业率保持在40%以上，城镇登记失业率控制在3%以下，2003年“4050”人员安置比例、购买公益性岗位比例位居全省第一。国有企业下岗职工基本生活费、企业离退休人员养老金社会化发放率和按时足额发放率均达到100%。提高了城镇居民最低生活保障实际补助水平，基本实现了动态管理下的应保尽保。解决了15.25万人饮水困难和1.36万农村贫困人口的温饱问题，启动了4.36万农村特困人口的经常性救助工作。完善和落实安全工作长效机制，安全生产形势明显好转，2003年度被评为国家级安全生产监督管理先进单位。[Page]

　　过去的五年，是我市精神文明建设取得新成效，民主法制建设进一步加强的五年。认真学习邓小平理论、“三个代表”重要思想，加强社会公德、职业道德和家庭美德教育，扎实推进精神文明创建工作，深入开展“热爱鹤壁、增辉中原”，“道德规范进万家”，学习靳月英、郑永立、马景富等先进人物活动，广大干部群众的精神风貌和城乡面貌发生新的变化。实施依法治市，广泛开展普法教育，公民的法律意识进一步增强。坚持依法行政，严格执行人大及其常委会通过的决定和决议，坚持并完善向人大报告工作、向政协通报情况制度，认真办理人大代表议案、建议171件，政协委员提案1037件。密切与工会、共青团、妇联等人民团体的联系，注意接受社会监督。积极推进政务、村务、厂务公开，努力扩大基层民主。认真做好信访工作，成为全省唯一没有信访工作重点管理县区的省辖市。加强社会治安综合治理，连续四年获得省综合治理先进市称号。深入开展“严打整治”斗争，加强社会防控体系建设，认真做好“扫黄”“打非”和“法轮功”人员教育转化工作，社会大局保持稳定。

　　各位代表，2003年是本届政府任期的最后一年。在遭遇非典疫情和自然灾害的情况下，我们迎难而上抓改革，一心一意谋发展，突出重点抓招商，加大力度上项目，经济社会发展成绩显著、硕果累累。与上年相比，全市生产总值增长13.5%，比全省平均水平高3个百分点；财政一般预算收入增长12.4%，其中市级增长19.3%；全社会固定资产投资增长31.5%，比全省平均水平高5个百分点；社会消费品零售总额增长11.2%；规模以上工业企业增加值增长17.7%，实现利润增长31.9%，经济效益综合指数提高15.4个百分点；外贸出口增长57.4%，比全省平均水平高16.8个百分点；城镇居民人均可支配收入增长12.9%，农民人均纯收入增长5.5%，分别比全省平均水平高3.9和5.9个百分点。全市生产总值、一般预算收入、全社会固定资产投资、社会消费品零售总额、规模以上工业企业增加值、经济效益综合指数、外贸出口增速均为五年来最高。特别是招商引资和大项目建设实现了跨越式增长，引进市外直接投资12.3亿元，其中工业领域超过11亿元，比上年增长6倍。可以说，我们实现了全面建设小康社会的良好开局，跨入了加速发展、再上新台阶的关键阶段。

　　以上成绩的取得，是省委、省政府和市委正确领导的结果，是市人大、市政协监督支持的结果，更是全市广大干部群众共同努力的结果。在此，我代表市人民政府，向全市广大工人、农民、知识分子、干部、驻鹤部队指战员、武警官兵及社会各界人士致以崇高的敬意！向关心、支持我市现代化建设的朋友们表示衷心的感谢！

　　五年来的实践给了我们许多有益的启示。一是必须坚持紧抓第一要务不放松。五年来，我们始终以经济建设为中心，把发展放在头等重要的位置，靠发展统一思想、凝聚人心、鼓舞斗志，靠发展解决前进中的困难和问题，各项工作都自觉服从服务于发展这个大局。二是必须坚持创新开拓，求真务实。五年来，我们始终站在全国、全省的大格局中审视自己，积极探索并不断完善体现时代要求、切合鹤壁实际的发展路子，并以此为指导，扎扎实实推进各项工作。由于思路清，工作实，全市上下加快发展的热情明显高涨，工作节奏明显加快，工作质量明显提高。三是必须坚持突出重点，寻求突破。五年来，我们始终抓住大项目建设、招商引资、深化改革、城镇化、非公有制经济、就业再就业等关系全市经济社会发展的重点工作，集中力量，实施突破，有力地带动了各项工作的全面推进。四是必须坚持团结协作，群策群力。五年来，市政府始终坚持市委的统一领导，自觉接受市人大、市政协及全市人民的监督，靠科学机制和正确导向调动广大干部群众干事创业的积极性，营造了团结融洽谋发展的良好环境，形成了和衷共济创大业的浓厚氛围。

　　我们也清醒地看到，与全国、全省先进地区相比，我市经济社会发展还存在较大差距，政府工作还有不少亟待改进和加强的地方。经济总量小，产业基础薄弱，地方财政困难。城镇居民人均可支配收入水平较低，部分群众生活还很困难。对一些地方的社会治安，群众还不太满意。部分政府工作人员思想守旧、满足现状，大局意识、责任意识不强，缺乏创新开拓、拼搏进取精神，工作质量和工作效率不高。经济发展环境还需要更进一步改善。对于这些困难和问题,我们必须有一个清醒的认识,在今后的工作中切实加以解决。

　　二、今后五年政府工作的[Page]

　　指导思想、奋斗目标及2004年工作建议

　　今后五年是我市全面建设小康社会的关键时期。当前，我们已经具备了乘势而上、加快发展的基础，是一个重要而难得的发展机遇期。面对新一轮更加激烈的竞争，我们必须以高度的紧迫感和责任感，进一步解放思想，更新观念，采取超常规的举措，付出百倍的努力，实现我市经济社会的跨越式发展，为实现中原崛起作出应有的贡献。

　　今后五年政府工作的指导思想是：以邓小平理论和“三个代表”重要思想为指导，坚持科学的发展观，按照“五个统筹”的要求，围绕“两个率先”奋斗目标，思想再解放，思路再创新，效率再提高，发展再加速。紧紧抓住发展第一要务，把发展经济作为政府执政为民的首要任务，大力推进工业化、城镇化和农业现代化。着力解决关系人民群众切身利益的突出问题，正确处理改革发展稳定的关系，推动经济社会全面、协调、可持续发展，实现社会主义物质文明、政治文明和精神文明共同进步。

　　按照上述指导思想和《鹤壁市全面建设小康社会规划纲要》，今后五年要努力实现全市生产总值年均增长11.5%以上；财政一般预算收入年均增长11%；全社会固定资产投资年均增长25%以上；社会消费品零售总额年均增长11%；引进市外直接投资年均增长30%；城镇居民人均可支配收入年均增长10%，农民人均纯收入年均增长6%；人口自然增长率控制在6‰以内；城镇化率达到47%；下岗失业人员再就业3.1万人，城镇登记失业率控制在4%以内。

　　实现今后五年的奋斗目标，开好头、起好步非常重要。根据省委经济工作会议、省十届人大二次会议和市委经济工作会议精神，今年我市经济社会发展的主要预期目标是：全市生产总值增长12%以上；财政一般预算收入增长9%以上；全社会固定资产投资增长35%以上；社会消费品零售总额增长11%以上；城镇居民人均可支配收入增长11%，农民人均纯收入增长6%；人口自然增长率控制在4.5‰以内；城镇登记失业率控制在4%以内。

　　今年要重点抓好以下工作：

　　(一)继续把招商引资放在经济工作的首位，达到高目标、实现新突破

　　继续把招商引资作为全市经济工作的重中之重，以工业为重点，以项目特别是大项目建设为载体，全方位、大力度、高效率开展工作。通过各级努力，确保引进市外直接投资32亿元以上，力争达到37亿元，其中工业领域22亿元以上，力争达到27亿元，在去年大幅度增长的基础上，继续保持数倍的增长。

　　壮大招商引资力量。市级、县区政府领导积极外出招商引资并联系具体项目。进一步加强市、县区招商引资队伍建设，使全市招商引资队伍形成有机整体，充分发挥职能作用。进一步加大对招商引资的投入和工作力度；注重发挥企业的招商引资主体作用；引导、组织社会各界关心、支持、参与招商引资，真正形成举全市之力招商引资的局面。

　　完善激励奖惩机制。实行招商引资进展情况定期汇报与督促制度。完善考核和奖励办法，扩大招商引资奖励资金规模，进一步发挥导向与激励作用。引导协调各县区、市直有关部门加大整体宣传推介鹤壁力度，不断提高我市的知名度，推进全市招商引资工作。

　　创新更具实效的招商引资方式。与国内外知名企业、投资机构、中介机构、科研机构、大专院校建立密切的合作关系，拓展招商引资渠道与方式。在香港、深圳、广州、上海、青岛等地设立招商引资窗口，以新的机制开展工作，特别是在吸引港资方面要取得积极进展。抓住全省加强豫沪合作的机遇，力求实绩。选择适当时机适当载体，组织几次有影响的招商活动。做好2004台胞国庆观礼团来鹤各项工作，积极争取和吸引国家工商联执委民营企业家等考察团，以及鹤壁籍台港澳同胞、海外侨胞前来参观考察、投资洽谈。完善制度和机制，搞好签约项目的协调服务、跟踪问效。

　　努力扩大招商引资领域。突出抓好工业领域的招商引资，力争在金属镁精深加工、水资源利用、食品加工、生物制药、汽车零配件、电子元器件、纺织等重点领域实现新突破。同时，加大农业、教育、卫生、文化、体育、旅游、外向型市场、新型商业业态等领域的招商引资力度。注意把引进资金、项目、技术与引进人才、智力结合起来，形成相互促进的良好局面。做好招商引资信息的收集、整理、交流，实现高效传递和共享。根据国家、省产业政策，重点围绕“三大基地”建设，不断储备和策划新项目、大项目，把前期工作做细做实，保证一批大项目能够直接运作实施。

　　(二)以“三大基地”建设为重点，加速推进工业化[Page]

　　科学、全方位地搞好策划，以招商引资为主要手段，以做大做强优势特色产业为主攻方向，强力推进大项目，带动小项目，进一步加快“三大基地”建设。规模以上工业企业增加值增长18%，实现利润增长8%，经济效益综合指数提高5个百分点。

　　加快建设资源型工业基地。基本完成电厂二期主体工程，争取三期工程上半年开工。加快热电联供项目推进速度。积极推进水泥厂三期工程建设，加大四期工程前期工作力度，力争年内开工。做好金属镁冶炼规模扩张与精深加工，水、电力资源开发利用，水泥、煤炭产业链条延长等工作，争取实现新突破。加快安鹤煤田开发，完成煤矿安全技改、综采机械化装备更新等项目。

　　加快建设先进制造业基地。天淇汽车模具生产线项目竣工投产。天鹤汽车模具扩产改造、飞鹤公司安全玻璃胶片项目开工建设。迈世通生物医药科技园项目一期工程投产。市中药厂、浚县中药厂GMP车间年内完工。天华科技园项目建成并投入运营。野战通信车项目竣工验收。同发公司3万锭精梳项目开工建设。同时,再谋划、引进、建设一批科技含量高、竞争能力强、吸纳劳动力容量大的新项目、大项目。

　　加快建设食品加工业基地。集中精力做大做强畜牧加工业，搞好畜产品规模化、标准化、产业化生产和精深加工，优化产品结构，打造安全食品品牌。抓好已确定的12个重点畜产品生产加工项目，争取通过招商引资再上一批新项目。畜牧加工业产值超过30亿元，增长20%以上。加快发展粮食精深加工，力争一家大型小麦加工企业落户我市。

　　强力推进重点项目建设。按计划、高质量推进盘石头水库建设。认真组织实施采煤沉陷治理项目，力争完成总投资的25%以上。公路建设力争完成投资4亿元，开工建设大海线、浚南线、东上线二级公路改建工程和7条国债资金县乡道路，完成6条续建国债资金县乡道路建设；新老区快速通道完成投资总量的40%；继续支持鹤濮高速公路建设，力争使通向山西省方向的高速公路项目前期工作取得积极进展。新区污水处理厂年底试运行。做好新区垃圾无害化处理工程争取国债资金工作和项目前期工作。积极推进医疗垃圾无害化处理项目。大力争取并管好用好国债资金，确保国债项目工程质量和资金使用效益。全年完成固定资产投资45亿元以上，力争达到50亿元以上；投资规模超千万元的在建项目超过100个，总投资规模超过150亿元。

　　(三)以新区建设为龙头，加快城镇化进程

　　坚持市、县区、重点镇三头并举，突出特色，加快新区建设，带动老区、县城及重点镇建设，培植产业支撑，完善城市功能，提高管理水平，实现一体化发展。全市城镇化率达到42%以上。

　　大力培育产业支撑。把发展产业作为聚集新区人气、壮大经济实力的基本途径，加快工业园区、高教园区、外向型市场和旅游业发展，带动其他社会事业项目建设。完善工业园区基础设施和服务体系，加快新区工业发展步伐。积极推进工贸学校和技校新校区建设，做好技师学院的申报争取及相关工作。高教园区招商引资力争取得突破。加快以特色批发市场为主的外向型市场建设，完成豫北粮食仓储物流交易中心仓库建设，建设豫北最大的优质小麦现货交易中心。争取丹尼斯超市落户新、老区。淇河综合开发取得实质性进展，保护和管理水平进一步提高，景区接待能力明显提高。浚县、淇县依托“两山”，加快旅游业发展。搞好旅游资源的整合、策划和包装，加大旅游促销投入，把中国优秀旅游城市的品牌喊响叫亮，争取旅游直接收入增长40%。

　　加强城市建设与管理。一是把新区建设推向新台阶，打造“钻石般的晶莹、火焰般的活力、田园般的美丽”的城市形象。完成、新开工道路21公里，城市框架拉大到38平方公里。摆放投资规模1000万元以上的项目25个，其中亿元以上的项目8个；开工建筑面积达到60万平方米，投资超过10亿元。完成王升屯村改造。全面启动大赉店村改造，其中黄河路以北部分基本完成。根据高教园区建设和新区南部城区拓展需要，启动相关村庄改造。迎宾馆投入使用。游泳馆竣工。温泉度假村项目完成招商引资。继续开展火车站三级升二级争取工作。高标准高品位建设、改造绿地16.7万平方米，使绿化美化成为我市城市的特色和优势。二是重视完善老区道路、供排水、供电、通讯等城市基础设施，加快鹿楼地带改造、规划与开发建设。三是规划实施新老区连接带开发。加快庞村镇产业聚集。完成高档住宅区规划，部分项目开工建设。四是加快两县县城和重点镇建设。严格控制零星建设尤其是两层以下住宅建设，推动住宅小区建设。策划实施经营城市工作。五是开展农村新社区建设试点。结合空心村治理，探索启动农村村庄改造，改善农民生活环境。六是切实提高城市管理水平。全面提高环卫、灯光、绿化、道路的管理标准。统筹考虑城市地下设施建设，实现管沟开挖规范管理。改革城市公厕管理模式，提高管理水平。治理影响市容的突出问题，加强背街小巷管理。在老区和县城逐步推行集中供热。深化户籍管理制度改革，完善落实有关政策，做好城镇迁入人口的社会服务，促进农民向城镇转移。[Page]

　　高起点高标准搞好规划。优选规划设计单位，高水平编制城市总体规划和专项规划。完成新区部分专项规划，对山城区、鹤山区总体规划进行修编，推动两县县城和重点镇规划的编制完善。推行规划公示制，加强规划审批和批后管理，严格按规划建设。

　　(四)以增加农民收入为核心，积极推进农业现代化

　　认真宣传贯彻中央、省增加农民收入，加快农业农村经济发展的方针政策，坚持以工业的理念发展农业，强力实施农产品加工带动，进一步提高农业产业化水平，促进农民增收和农业农村经济全面发展。

　　加快培育壮大龙头企业，推进农业产业化经营和结构调整。依托优势农产品资源，大力扶持、培育加工龙头企业和种植、养殖大户，引导、支持加工龙头企业加快与市外大企业联姻，扩大经营规模，拉长产品和产业链条，提高市场竞争力，增强辐射带动能力。策划中原畜产品物流中心。抓好善堂镇、新镇镇、钜桥镇农村专业合作组织试点，加强农民经纪人队伍建设。抓住国家、省推进优势农产品产业带建设的机遇，加快优质畜产品、优质粮食生产加工基地建设。突出畜牧业优势，稳定蛋鸡、肉鸡生产，积极发展瘦肉型猪生产，大力发展牛、羊和奶业生产。畜牧业产值达到30亿元，占农业总产值的比重达到62%。稳定粮食生产，加快发展优质、高效、生态、安全农业。力争优质小麦播种面积达到90万亩，优质玉米达到30万亩。新增优质经济林2万亩。做好“淇河三珍”品牌开发。

　　搞好劳务输出，增加农民务工收入。引导、支持农民外出务工，加大农民工职业技能培训力度，做好外出务工人员的组织、协调和管理工作，在我市外出务工人员相对集中的地方设立办事处，积极收集用工信息，为务工人员提供维权服务。全年新增劳务输出7万人，累计达到13万人。

　　深化农村改革。深化农村税费改革，降低农业税税率3个百分点。深化粮食流通体制改革，确保将粮食直补资金兑现给粮农。创新农村金融体制，加快农村信用社改革。推进土地征用制度改革。

　　提高农产品质量安全水平。抓好国家食品安全综合示范区项目。做好高致病性禽流感、口蹄疫等动物疫病防治工作,加快建设动物疫情监测预报和防疫体系。整合省畜产品检验检测中心鹤壁分中心、市农产品质量检测中心、市粮油质量检测中心,尽早投入使用。

　　加强农业和农村基础设施建设。发展节水灌溉5万亩。完成2.4万亩中低产田改造，使6万人受益。完成人工造林11.3万亩。治理水土流失45平方公里。空中降水工程覆盖面由45%提高到90%以上。

　　发展壮大县域经济。认真贯彻落实中央、省发展壮大县域经济的决策部署，以工业化为主导，以招商引资和项目带动为抓手，坚持扶持、放权、让利，引导和督促两县发展优势产业和特色经济，尽快增强经济实力。今后五年，两县综合经济实力在全省排名每年都要前移至少一个位次。注重淇滨区、山城区、鹤山区产业和生产要素聚集，尽快做大做强各具特色和竞争优势的产业。

　　(五)进一步改善发展环境，实现非公有制经济发展新突破

　　以创造更高层次的发展环境为目标，高度重视环境治理工作，加强对各级各部门特别是基层执法人员的教育管理，督促各级各部门认真落实并不断完善服务非公有制经济的政策和措施，真正做到寓管理于服务之中。制定明确的惩处办法，严肃查处破坏发展环境的各种案件，及时处理并公开曝光，使我市的发展环境得到进一步的明显改善。

　　坚持把发展非公有制经济与招商引资、国企改革、发展县域经济等结合起来，认真贯彻落实国家、省出台的优惠政策，进一步完善支持非公有制经济发展的政策措施。继续安排专项资金，支持中小企业信用担保中心建设。筹建投资担保公司，加大融资支持力度。引导、支持非公有制企业成立互助性担保机构。增加奖励基金，加大奖励非公有制企业力度。支持和鼓励有条件的非公有制企业加快建立现代企业制度，努力培育上规模的非公有制企业。引导监督非公有制企业依法经营，保障职工的合法权益。力争非公有制经济增加值增幅明显高于去年。

　　(六)把改革贯穿各项工作始终，努力推进体制创新和机制创新

　　积极稳妥推进国有企业改革。坚持一厂一策，协同攻坚，上半年基本完成工业企业改革任务，其他企业争取10月底前基本完成改革任务；需要保留国有资本的政策性国有企业，也要转换机制，严格管理，提高效率。建立企业改革专项基金，促进重点难点企业改革。继续把企业改革与招商引资、发展非公有制经济等结合起来，努力引进实力雄厚、业绩良好的战略合作者。认真贯彻落实国家关于企业改革的政策法规，妥善解决职工安置、资产处置、债权债务处理等问题，维护国家、企业、资产所有者特别是广大职工的合法权益。同时，重视挖掘现有企业潜力，通过搞好企业家队伍培训等服务，促使企业发挥优势，加快发展。[Page]

　　深化城市建设和管理体制改革。规范完善国有土地储备制度并向两县延伸，以新区、淇河两岸、快速通道和大白线两侧、鹿楼地带为重点，新储备各类用地1000亩以上，实现土地收益1亿元以上。巩固土地市场清理整顿成果，搞活房地产二级市场，积极培育房地产业。科学规范地推进城市公用事业特许经营。探索建立有效的城市综合执法新机制。

　　加快投融资体制改革。加大创新力度，改善融资手段，拓宽融资渠道。充分发挥金融在经济社会发展中的重要作用，引导鼓励金融机构大力支持优势企业和有潜力企业，支持县域经济和非公有制经济加快发展。密切银政、银企合作，切实维护金融债权，加快优化金融资产结构，努力建设金融安全区。积极协助鹤壁煤电股份公司做好上市工作，引导、帮助一批有实力、有潜力的企业开展上市前的准备工作。有效运作市经济发展建设投资公司，增强市场化融资和运作能力。推动城市信用社向城市商业银行方向发展。成立创业投资公司。积极争取、有效使用打捆授信资金。整合政府投资资源，推动已建成的国有投资项目依法转让产权或经营权，盘活资产存量，实现滚动发展。探索有效的方法和机制，推动民间投资增长。

　　推进社会事业改革。深化办学体制改革，大力发展民办教育。深化文化体制改革，创新文化管理体制和运行机制，积极推进文化产业发展。加快医疗机构体制改革和医疗卫生资源整合与发展步伐，积极推进乡级医疗机构市场化改革。

　　深化行政管理体制和事业单位改革。上半年完成市、县区政府机构改革任务。全面完成县区事业单位人事聘用制度改革。通过编制调整，优化行政事业单位人力资源配置，提高政府工作效能。从各县区和市直分别选择3-5个事业单位作为体制改革试点。

　　深化财政改革。加快财政支出管理制度改革，合理调整支出结构，充分调动并妥善运用各方面财力，确保重点支出。坚持依法治税，应收尽收。规范完善政府债务资金管理，安排偿债准备金并纳入预算。加大向上争取资金力度，确保比去年有更大突破。

　　(七)切实解决好关系人民群众切身利益的问题，满腔热忱为群众办实事

　　实施积极的就业政策，完善就业服务体系，加强就业技能培训、职业介绍和就业指导。完成“两个确保”任务，做好失业保险、城镇居民最低生活保障及农村特困人口救助工作。努力扩大医疗保险覆盖面。加大劳动保障执法监察力度，切实维护劳动者合法权益。改善农民进城就业环境，努力解决克扣和拖欠农民工工资问题。坚决治理教育乱收费。严厉打击制售假冒伪劣食品药品、医疗器械、农资等违法犯罪行为。依法做好失地农民和城镇拆迁户补偿、安置工作。

　　坚持不懈抓好安全工作。严格执行《安全生产法》，建立完善安全生产长效机制，实行安全生产许可证制度，加快构建市、县、乡、村四级监管网络。继续深化小煤矿、非煤矿山、危险化学品、民爆器材和烟花爆竹、道路交通、公共聚集场所消防、旅游、建筑施工等专项整治。做好中小学危房改造、水上安全等工作。坚决查处各类安全事故，依法追究有关人员责任。

　　从群众最关心、与群众切身利益联系最紧密的问题抓起，年内着力抓好以下几个方面的实事：

　　1、对1万多户农村特困户实施分类救助。巩固温饱人口5200人，解决贫困人口1100人。对义务教育阶段特困生免杂费、免书本费、补助住宿生生活费，保证不因家庭困难而辍学。免费对5000名农民进行就业培训。解决农村2万人饮水困难。

　　2、新增城镇就业1.5万人，安置下岗失业人员再就业7000人。企业职工参加工伤保险人数新增6万人，达到8万人。

　　3、取缔新区供热区取暖燃煤小锅炉。完善新区城市排水系统，实现棉丰渠渠水变清并实施进一步改造。启动二支渠生态建设工作。明显改观老区环境卫生。新增街头游园2-3处。山城区西环线建成通车，牟山大道全面整修。

　　4、新区、老区物业管理覆盖面分别超过90%和40%，并进一步提高物业管理水平。选择7个社区作为示范，推进全市社区建设工作。

　　5、建成120急救指挥中心，健全覆盖全市的突发公共卫生救治网络体系。完成市传染病医院门诊楼和病房楼、两县医院传染病科和中心乡卫生院传染病门诊建设。完成2所中心乡卫生院建设。

　　6、鹤壁高中、二中新区分校开始招生。青少年学生校外活动中心投入使用。普通高中招生比去年增长14%以上，满足社会需求。

　　7、大力开展治安乱点整治工作，明显改观治安状况。发布市及两县消防规划，完成327个市政消火栓建设。

　　8、开展服务“三农”集中活动并建立长效机制，使受益人数达到100万人次以上。市文化中心9月份对外开放。[Page]

　　(八)加强社会主义精神文明建设，全面发展各项社会事业

　　深入学习宣传邓小平理论和“三个代表”重要思想。认真贯彻《公民道德建设实施纲要》。积极开展群众性精神文明创建活动。普及科学知识，弘扬科学精神。倡导科学、健康、文明的生活方式。加快社会信用体系建设。搞好农村、社区和企业等基层文化建设，加强文化市场管理，对全市网吧等互联网上网服务营业场所重新审核登记，从严把关,严格管理。进一步发展广播电视、新闻出版、文学艺术事业。

　　大力培养、积极引进、合理使用各类人才。做好申报国家高新技术特色产业基地工作。启动科技研发园建设。加强农村科技推广体系建设，提高科技创新、储备和转化能力。巩固提高“两基”水平，保证每个小学适龄儿童入学，保证给每个初中学龄人口提供就学机会。以职业技术学院为主体，突出重点专科，办出特色，办出水平，做好专升本工作。利用全市各级各类职教基地，围绕产业发展需求，搞好实用技术人才培养培训。鹤壁高中力争创建成省级示范性普通高中。淇县高级中学一期工程完工。搞好农村中小学远程教育试点工作。积极倡导中小学教育的科学管理，开展校际帮扶，提高全市整体教学水平。健全突发公共卫生事件和重大传染病应急处置机制，完善公共卫生体系，进一步强化公共卫生职能。做好重点综合医院的改革与发展工作。支持上规模上水平的民营专病专科医疗机构发展。搞好农村初级卫生保健体系建设。巩固提高新型农村合作医疗试点工作。每月公布食品卫生监测结果和消费警示。按照便民、实用、持久原则新增一批专项体育设施，举办市第八届运动会，深入开展全民健身活动。组织好全市第一次经济普查。做好民族工作，依法管理宗教事务。稳妥推进殡葬改革。关心、支持驻鹤部队和民兵预备役建设，妥善安置转业退伍军人。深入开展拥军优属和军民共建活动，进一步密切军政军民关系。

　　坚持科学的发展观，走可持续的发展道路。加强人口和计划生育工作，保持人口自然增长率低于全省平均水平。注重资源保护、节约和再生利用，大力推行清洁文明生产方式，依法关闭产品质量低劣、浪费资源、污染严重的企业。积极实施地下水保护行动计划。争取上级支持，搞好卫河水污染治理。每日公布市区空气质量。强化环保执法和生态环境监管。

　　(九)加强民主法制建设，维护社会大局稳定

　　加强普法宣传教育，在全社会营造学法、懂法、守法的良好环境。自觉接受人大及其常委会的监督，认真执行人大及其常委会通过的决定和决议。加强与人民政协的协商，自觉接受政协的民主监督。切实办理好人大代表议案、建议和政协委员提案。广泛听取各民主党派的意见，充分发挥人民团体的桥梁纽带作用，接受社会监督。完善村民自治和城市居民自治，尊重基层和群众的民主权利。坚持和完善职工代表大会和其他形式的企事业单位民主管理制度，保障职工的合法权益。

　　进一步创新和改进信访工作，集中力量化解信访积案，积极预防和及时处理新的信访案件，妥善解决群体性事件。落实社会治安综合治理各项措施，狠抓基层基础工作，推进社会治安防控体系建设。建立健全经常性的严打机制，始终保持对各种犯罪的高压态势。

　　(十)大兴求真务实之风，加强政府自身建设

　　坚持执政为民，建设责任型政府。树立科学的发展观和正确的政绩观，追求符合经济发展需要、符合广大群众愿望的实实在在的政绩。完善并严格实行目标管理责任制，对每项工作细化量化,加强督查指导，严格考评奖惩，保证市委、市政府决策部署的贯彻落实。全面启动效能监察和行风评议，使解放思想、创新开拓，拼搏实干、俯首为民，尽职守责、争创一流成为各级干部的自觉行动，形成干大事、创大业，以发展快慢论英雄的浓厚氛围。

　　坚持依法行政，建设法治型政府。认真贯彻实施《行政许可法》，全面清理不符合规定的行政许可，加快配套制度建设。严格按照法定权限和程序行使权力、履行职责。强化行政执法监督检查，建立行政执法责任制和行政过错追究制。

　　坚持务实高效，建设服务型政府。加快推进电子政务，完善政府网站系统，构建行政审批平台，改进政府工作流程。大兴调研之风，切实精文简会。大力加强公务员队伍能力建设，对全市公务员进行全面系统的业务培训。加强服务网络建设，尽快形成市、县、乡便民服务网络。加强对部门中层干部、基层队伍的管理，使直接面向广大群众的基层组织、基层单位切实转变工作作风，为群众提供优质高效服务。[Page]

　　坚持政务公开，建设透明型政府。政府规范性文件和有关行政措施、政务活动等，凡适宜公开的向社会公开，公共事业领域和具有行政职能的事业单位也要推行政务公开。充分发挥新闻媒体、政府网站的作用，建立广大群众、企业经营者与政府部门的直接对话交流机制。通过新闻媒体、政府网站有选择地公布政府有关工作进展情况。市、县、乡都要制定政务公开监督制约制度，公众意见建议收集、解决、反馈制度，确保公开质量。

　　坚持廉洁从政，建设廉洁型政府。认真落实党风廉政建设责任制，健全管理监督机制。把解决群众反映的突出问题作为反腐倡廉工作的重点，从严治标，着力治本。坚持从严治政，赏罚分明，严肃查处违纪违法案件。全体政府工作人员特别是各级领导干部，都要继续保持谦虚谨慎、不骄不躁的作风，继续保持艰苦奋斗的作风。

　　各位代表！新形势新目标催人奋进，鹤壁的事业任重道远。我们坚信，在省委、省政府和市委的正确领导下，新一届政府一定能够与全市人民一道，与时俱进，拼搏实干，以新的精神状态、新的工作面貌，在实现“两个率先”的征途上阔步前进，创造鹤壁更加美好的明天！
